# Lymphocutaneous Sporotrichosis

**DOI:** 10.4269/ajtmh.21-1212

**Published:** 2022-01-24

**Authors:** Alvaro Schwalb, Paloma M. Carcamo, Carlos Seas

**Affiliations:** ^1^ Instituto de Medicina Tropical Alexander von Humboldt, Universidad Peruana Cayetano Heredia, San Martín de Porres, Lima, Peru;; ^2^ School of Medicine, Universidad Peruana Cayetano Heredia, San Martín de Porres, Lima, Peru

**Figure 1.**
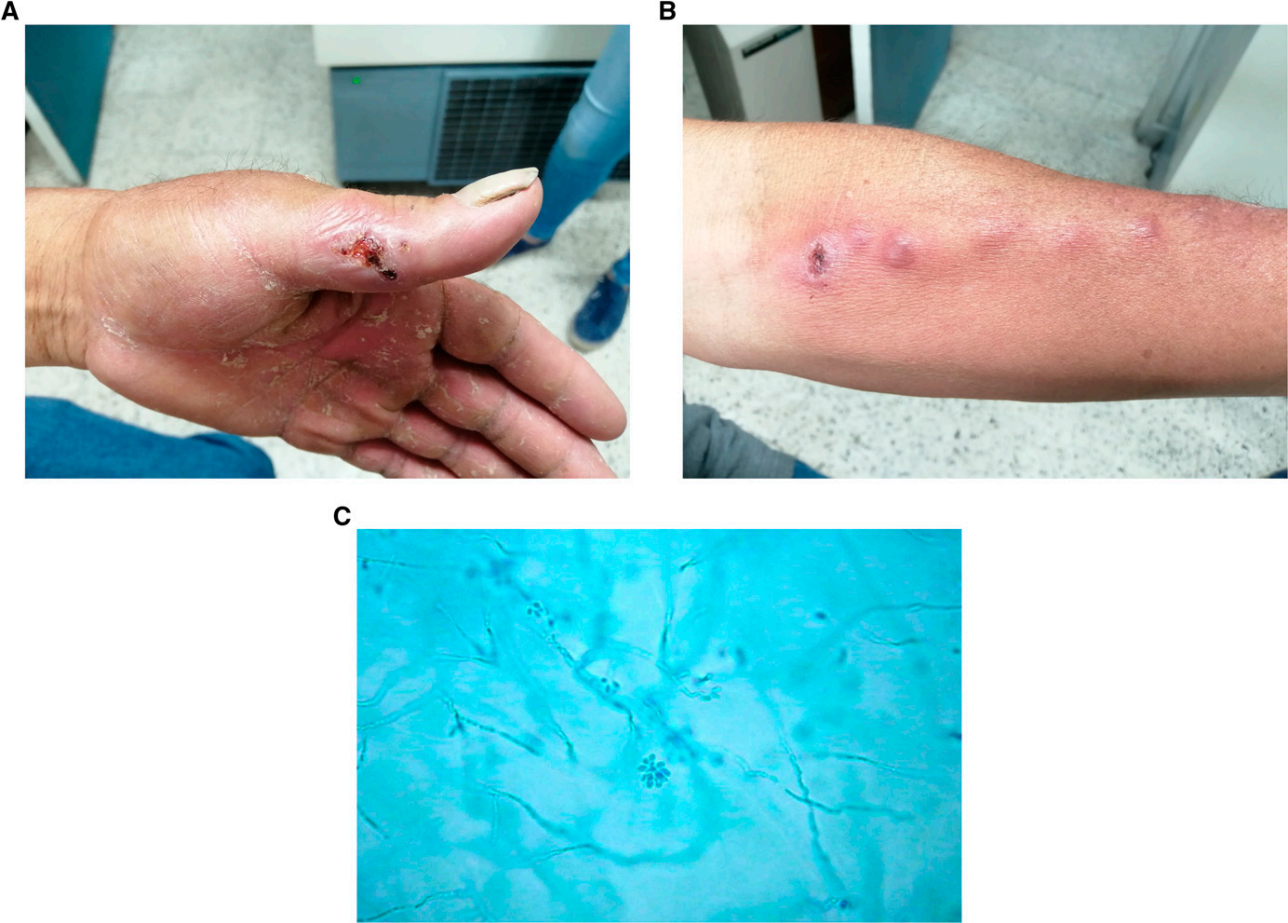
(**A**) Small, ulcerated lesion with surrounding erythema and edema in the left thumb where minor trauma with a spike occurred. (**B**) Multiple erythematous subcutaneous nodules following lymphatic spread in anterior left forearm. One nodule appears crusted. (**C**). *Sporothrix schenckii* colonies showing branching narrow hyphae and the characteristic bouquet-like appearance of the microconidia. This figure appears in color at www.ajtmh.org.

A 58-year-old male farmer from the Ancash region in the Peruvian highlands presented to the outpatient clinic with an ulcerated lesion on the left thumb and several nodular lesions on the left forearm. He sustained minor trauma from a wood splinter in his left thumb a month before presentation. Later, the wound ulcerated and started to drain serous fluid (Figure 1A), with the subsequent appearance of multiple small, erythematous, and painless nodules in his left forearm (Figure 1B). Some had undergone spontaneous suppuration with ensuing crusting. Culture of the aspirate from the nodular lesions was positive for *Sporothrix schenckii* by demonstrating dimorphism on enriched media.^1^ The colonies showed the characteristic bouquet-like microconidia in microscopy (Figure 1C). The patient was started on itraconazole 200 mg daily for 3 months.

Lymphocutaneous spread is the most common clinical manifestation of sporotrichosis, with most cases occurring among individuals with occupational exposure to the *S. schenckii* fungus.^2^ Vulnerable occupations generally include farming, gardening, beekeeping, and carpentry.^3^ Furthermore, sporotrichosis is frequently found in the Andean provinces of Peru. One province in particular—Abancay in Apurimac—has been identified as a hyperendemic region for sporotrichosis, with a mean annual incidence of 98 cases per 100,000 inhabitants.^4^ Ancash, the region in which our patient lives, only reports a few isolated cases per year.^4^ Although lymphocutaneous sporotrichosis is not usually life-threatening, lesions will not resolve without treatment.^5^ With appropriate treatment, they usually remit within 1 month, although factors such as suboptimal dosing of antifungals, nonadherence to treatment, or use of antacid medications may extend the duration of illness.^6^ The treatment outcome for this patient is unknown as his follow-up was done at the local medical center in his hometown.
